# Explaining variance in self-efficacy among adolescents: the association between mastery experiences, social support, and self-efficacy

**DOI:** 10.1186/s12889-023-16603-w

**Published:** 2023-08-30

**Authors:** Annette Løvheim Kleppang, Anne Mari Steigen, Hanne Søberg Finbråten

**Affiliations:** 1 Department of Public Health and Sport Sciences, Faculty of Social and Health Sciences, Inland Norway University of Applied Sciences, Elverum, Norway; 2 Department of Health and Nursing Science, Faculty of Health and Sport Sciences, University of Agder, Kristiansand, Norway; 3 Department of Health and Nursing Sciences, Faculty of Social and Health Sciences, Inland Norway University of Applied Sciences, Elverum, Norway

**Keywords:** Adolescents, Mastery experiences, Self-efficacy, Social support

## Abstract

**Background:**

Self-efficacy has been identified as an important health-promoting factor for both physical and mental health. Previous studies have examined self-efficacy as a moderating factor between negative psychosocial influences and various outcomes, e.g., life satisfaction and stressors. There is, however, limited knowledge about factors that strengthen self-efficacy. The aim of this study is to examine the association between mastery experiences, social support, and self-efficacy among adolescents in secondary schools in Norway.

**Methods:**

This study is based on cross-sectional data from the Ungdata surveys conducted in eastern part of Norway in 2021. The sample comprises 9,221 adolescents aged 13–16. Sequential multivariate linear regression was conducted to explore the association between mastery experiences, social support, and self-efficacy.

**Results:**

The final model (Model 3) explains 25% of the total variance in self-efficacy. The indicators concerning mastery experiences – defined here as the personal experience of success – explain more of the observed variance in self-efficacy than the other independent variables (change in R square = 10.7%). The items ‘felt mastering things’ ‘and ‘felt useful’ make the strongest and most significant contributions to the variance in self-efficacy in the final model (β = 0.25, *p* < 0.001 and β = 0.16, *p* < 0.001, respectively), followed by the variables ‘support from friends’ and ‘parental support’ (β = 0.06, *p* < 0.001 an β = 0.06, *p* < 0.001).

**Conclusions:**

Mastery experiences are potential sources for creating and strengthening self-efficacy. Awareness of the health-promoting potential in (strengthening) self-efficacy among adolescents is important. Additional research is needed to further explore these associations.

## Background

Increasing attention has been paid in recent decades to mental health in adolescents. Both globally and nationally several studies have reported an increase in mental health problems, such as depression and anxiety among young people [1–4]. Identifying risk factors for mental health problems is essential, but it is important also to examine factors that potentially strengthen and promote mental health in adolescence.

Adolescence is a period of life characterized by a variety of physical, psychological, and social changes and challenges. This period can be especially challenging for individuals who experience psychological risk factors such as exposure to trauma, low self-esteem, hopelessness, or associations with negative peer groups [5]. However, adolescence also brings opportunities to build certain strengths and thereby support positive outcomes [5, 6], and most people go through this developmental stage without any trauma or problematic challenges [7]. From the outset, therefore, research should take a salutogenic approach by focusing on factors that support health and wellbeing, not solely those responsible for poor mental health [8].

Self-efficacy is described as a positive or salutogenic psychological factor – one that potentially protects or buffers against negative psychological influences [9]. The concept was originally developed by Bandura [10], as part of his social cognitive theory, and refers to a person’s belief in their coping abilities. According to a previous study, self-efficacy reduces risk-taking behaviours and has a positive influence on health decisions among adolescents [11]. Higher self-efficacy is also linked to higher resilience [12], which can influence adolescents’ perception of life satisfaction and may help them cope with normative stressors [13]. Jerusalem and Hessling [14] have concluded that self-efficacy is an important health-promoting factor – for both mental and physical health – and can be systematically strengthened through interventions in school. It is also found to have a strong relationship with career adaptability and could therefore be an important resource for adolescents who have to make educational choices [15]. Moreover, self-efficacy is an important concept in different academic contexts and has been studied in relation to specific subjects such as science, mathematics, and reading [16–18].

Bandura refers to self-efficacy as situation-specific; in other words, it is possible to have high self-efficacy in one area and less in another. This has led to numerous different scales intended to measure self-efficacy in various contexts [19, 20]. Schwarzer and Jerusalem [21] have argued for a more generalized approach to self-efficacy, in which mastery experiences are the most important contributing factor. Bandura [10] has also described how, once established, self-efficacy tends to generalize to other situations, though most predictably to activities similar to those where it first took root. After a robust degree of self-efficacy is established, the impact of failures seems to be reduced [10].

Bandura [10] argues that personal self-efficacy derives from four principal sources: performance accomplishments, vicarious experience, verbal persuasion, and emotional arousal. Performance accomplishment is related to what are known as mastery experiences. Mastery experiences are seen as essential to develop a strong degree of self-efficacy [11, 22]. In particular, personal experiences of mastery are especially influential for developing self-efficacy in adolescents [10, 21]. In addition, vicarious experiences via social models might also be important, whereby seeing close friends or fellow students succeed in mastering difficult tasks may help strengthen one’s sense of self-efficacy. Furthermore, verbal persuasion in the form of verbal backup from others – encouraging and believing in the individual – is also described as important [10]. Lastly, Bandura identifies emotional arousal as another constituent source that can affect perceived self-efficacy in coping with threatening situations. Such emotions can range from an elevated heart rate to feelings of anxiety – such symptoms might signal that one lacks skills to cope. Hence, having fewer emotional symptoms may help strengthen self-efficacy in a given situation [10, 22]. These other sources of self-efficacy are seen as having a weaker effect than own performance and mastery experiences [22].

Adolescent development can take place in a range of different social contexts. Family, school, and peers are social settings where self-efficacy may be strengthened at this time [22]. Social support and connections to other people are fundamental for human beings [23], who live in social contexts and are influenced by the social relationships they are engaged in. Social support provides humans with emotional-, esteem-, informational-, and instrumental support [24, 25]. Strong connections with other people and social systems are therefore found to be beneficial for the mental health of adolescents (those aged 12–15) [26], while social support is seen as an important salutogenic factor, both as a buffer and for its direct effect on mental health [27, 28]. The correlation between social support and self-efficacy in adolescents is previously found to be moderate [29].

Most previous studies have examined self-efficacy as a moderating factor between negative psychosocial influences and various outcomes [13, 30]. For example, Moksnes, Eilertsen, Ringdal, Bjørnsen and Rannestad [13] have found that self-efficacy moderates the association between stressors and life satisfaction. Self-efficacy is an important health-promoting factor [9], and a high level of self-efficacy is related to a better health-related quality of life [31, 32]. Hence, it is important to strengthen self-efficacy to promote and maintain both mental and physical health [33].

On the other hand, far less is known about factors that strengthen self-efficacy. It is important not merely to examine the role of self-efficacy as a moderating factor. We also need more knowledge about what potentially strengthens self-efficacy. Acquiring this knowledge is important for working with health promotion strategies among adolescents. Based on Bandura’s theory on self-efficacy and the importance of social support in adolescence, the aim of this study is to examine the association between mastery experiences, social support, and self-efficacy among adolescents in Norway.

## Materials and methods

Data from the national Ungdata surveys in 2021 [34] form a basis for this study. The Ungdata surveys are an annual, representative cross-sectional studies including adolescents from grades 8 to 13 (ages 13–19). All municipalities in Norway are invited to participate and are recommended to participate every third year. Adolescents are asked about different aspects of their lives, such as health and well-being, school issues, and relationships with family and friends [4]. The Ungdata surveys are conducted by Norwegian Social Research (NOVA) at Oslo Metropolitan University in collaboration with the Regional Drug and Alcohol Competence Centre (KoRus) in Norway. The surveys consist of a mandatory section, but also optional parts that the municipalities might choose to include. The general self-efficacy scale (GSES) is included in one of the optional parts.

In the current study, the analyses are based on data from 9,221 adolescents in lower secondary schools (aged 13–16) from 45 municipalities in the eastern part of Norway. Data were collected during the first half of 2021. Participation was voluntary and based on informed consent. The parents of participants under 16 years of age had the right of reservation and could withdraw their children from the study. The response rate among participants in lower secondary school was 87 percent [35].

The study was administered as a web-based survey at school during school hours. A teacher or an administrator was present to assist the participants if they had any questions. The analyses are based on anonymous data: the Ungdata surveys are conducted in line with the Declaration of Helsinki, and the privacy protection of the respondents is approved by the Norwegian Agency for Shared Services in Education and Research (Sikt).

The present study was approved by the Institutional Ethics Review Board at Inland Norway University of Applied Sciences (protocol code 21/01894).

### Self-efficacy

In the Ungdata surveys, self-efficacy is measured using five items derived from the 10-item version of the GSES [36, 37]. Respondents are asked how true the following statements are, according to their experience: ‘I always manage to solve difficult problems if I try hard enough’ (Item 1); ‘If someone opposes me, I can find the means and ways to get what I want’ (Item 2); ‘I am confident that I could deal efficiently with unexpected events’ (Item 3); ‘I can remain calm when facing difficulties because I can rely on my coping abilities’ (Item 4); ‘If I am in trouble, I can usually think of a solution’ (Item 5). A four-point rating scale is applied to each of these items: not at all true (1), hardly true (2), moderately true (3), and exactly true (4).

The five-item version has been found to have an acceptable fit with the unidimensional polytomous Rasch model and sufficiently high reliability, with a person separation index and a Cronbach’s alpha of 0.80 and 0.88, respectively, when analyses are conducted on data from lower secondary schools [38]. For the analyses in the present study, the self-efficacy variable was transformed into logit values using the RUMM2030Plus statistical package. Hence, the analyses were performed based on person-location estimates of self-efficacy and can be considered a continuous variable.

### Independent variables

For the purposes of our study, we have included variables about mastery experiences and social support. In addition, we have included sociodemographic variables such as gender, school level, parents’ highest completed level of education, and the adolescents' perception of their family economy. Parents’ education was measured by asking: ‘Are your parents educated to university or university college level? Responses were categorized as: ‘none of them (1)’, ‘yes one of them (2)’ or ‘both parents (3)’. Family economy was measured using the following question: ‘Financially, has your family been well off, or badly off, over the past two years?’ This was categorized as follows: ‘we have been well off the whole time (1)’, ‘we have generally been well off’ (2), ‘we have neither been well off nor badly off (3)’, ‘we have generally been badly off (4)’, ‘we have been badly off the whole time (5)’. The family economy variable was recoded to ‘good (3)’, ‘neither bad nor good (2)’, and ‘bad economy (1)’.

Mastery experiences were measured using two separate variables: ‘felt useful’ and ‘felt that you are mastering things’. Both variables offer five response options: always (1), often (2), sometimes (3), seldom (4), and never (5). The response scale was reversed in the analyses, where higher score indicating better mastery experiences.

Social support was measured using three variables (response options in parenthesis): i) ‘My parents are interested in my life’ (1-very true, 2-quite true, 3-not very true, 4-not at all true); ii) ‘My teachers care about me’ (1-totally agree, 2-somewhat agree, 3-somewhat disagree, 4-totally disagree); and iii) ‘I have at least one friend I can completely trust and can confide in about everything’ (1-yes, definitely, 2-yes, I think so, 3-I don’t think that, 4-I do not have any people nowadays that I can call friends). The response options were reversed in analysis, with higher scores implying better social support.

### Data analysis

Independent *t*-tests were conducted to study differences in self-efficacy across gender and other dichotomized variables. When studying differences in self-efficacy across groups, a one-way analysis of variance (ANOVA) was used. Sequential multivariate linear regression was conducted to explore the association between self-efficacy, social support, and mastery experiences, controlled for sociodemographic variables. The independent variables were entered in three sequential steps. Sociodemographic variables were entered in the first model, whereas items about social support were added in the second model. In the final model (Model 3), the independent variables about mastery experiences were entered in addition to the previously mentioned independent variables. By introducing the independent variables in steps, we could assess the association between the dependent variable and each independent variable controlled for the previously entered variables. Adjusted R square was used to assess the model fit, whereas R square change was used as an indicator for the contribution of newly entered independent variables [39]. The standardised β coefficient with p-value gives information about the unique contribution of each variable. Statistical significance was assumed at *p* < 0.05.

Initial analyses were conducted to assure the requirements for linear regression. The log-transformed self-efficacy data were assessed for normal distribution, linearity, and homoscedasticity. The independent variables were controlled for multicollinearity. The independent variables were in general weakly correlated, but for the items ‘felt useful’ and ‘felt that you are mastering things’ the correlation was 0.74. However, the variance inflation factor was 2 or lower for all variables.

Interaction analysis was applied to examine the influence of gender and grade level on the strength of the relationship between social support (support from friends, teachers and parental support) and self-efficacy. Possible interaction effects were examined using the likelihood ratio test (LR test), comparing models with and without interaction terms. The main effect model included family economy, parents’ education, gender, grade level, social support (support from friends, teachers and parental support) and mastery experiences (‘felt useful’ and ‘mastering things’) as independent variables and was tested against models including the following interactions: gender*support from friends, gender*support from teachers, gender*parental support, grade level*support from friends, grade level*support from teachers and grade level*parental support. A parallel interaction analysis was carried out for mastery experiences (‘felt useful’ and ‘felt that you are mastering things’) and self-efficacy. The incremental change in log-likelihood between the main effect models including interactions was not significant. Thus, this implies that the fit was not improved when applying interaction models. Therefore, in the results section, only the main effect model is presented.

Statistical analysis was performed using the Statistical Package for Social Sciences (SPSS), version 26 (IBM Corporation).

### Missing values

As the self-efficacy raw scores were transformed to person-location estimates, there were no missing values for the dependent variable, as the RUMM software handles missing data using full information maximum likelihood.

## Results

Table 1 shows the characteristics of the sample and self-efficacy across levels of person factors, social support, and mastery experiences.

**Table 1:** Sample characteristics and self-efficacy across levels of person factors, social support, and mastery experiences

Characteristics	n (%)	GSE mean (SD)	*P* value
Number of participants	9,221	1.15 (2.02)	
Females	4,564 (50.6)	0.75 (1.88)	< 0.001
Males	4,461 (49.4)	1.58 (2.04)	
*Age cohort*			
8th grade secondary school	2,811 (32.8)	1.11 (2.07)	0.071
9th grade secondary school	2,933 (34.2)	1.12 (2.02)	
10th grade secondary school	2,827 (33.0)	1.22 (2.00)	
*Family economy*			
Good economy	7,306 (80.6)	1.34 (1.99)	< 0.001
Nor bad nor good economy	1,427 (15.7)	0.52 (1.87)	
Bad economy	328 (3.6)	0.05 (2.26)	
*Parents with higher education*			
No parents with higher education	1,215 (14.3)	0.98 (2.15)	< 0.001
One parent with higher education	2,900 (34.1)	1.09 (1.96)	
Both parents with higher education	4,387 (51.6)	1.32 (2.09)	
*Social support*			
Support from friends	8,211 (89.3)	1.23 (1.99)	< 0.001
Less support from friends	983 (10.7)	0.55 (2.13)	
Parental support	8,359 (91.4)	1.24 (1.99)	< 0.001
Less parental support	786 (8.6)	0.24 (2.11)	
Support from teachers	7,918 (86.5)	1.25 (1.97)	< 0.001
Less support from teachers	1,232 (13.5)	0.60 (2.21)	
*Mastery experiences*			
Felt useful	6,974 (76.8)	1.53 (1.92)	< 0.001
Felt less useful	2,104 (23.2)	-0.09 (1.85)	
Felt that you are mastering things	7,234 (80.4)	1.50 (1.91)	< 0.001
Felt that you are not mastering things	1,767 (19.6)	-0.23 (1.87)	

*GSE* General self-efficacy. Missing values represented 7.0% for age, 2.1% for gender, 1.7% for family economy, 7.8% for parents with higher education, 0.3% for support from friends, 1.0% for parental support, 0.8% for teachers’ support, 1.6% for ‘felt useful’ and 2.4% for ‘felt that you are mastering things’. Differences across dichotomized person factors were analysed using the independent sample t-test. Differences in GSE across groups was analysed using one-way analysis of variance (ANOVA)

A total of 9,221 adolescents from lower secondary school responded to the survey. The age distribution in the sample was similar across most categories, and the proportion of males and females was approximately equal. Among half of the adolescents, both parents had higher education and most of them reported a good family economy. The one-way ANOVA indicated statistically significant differences in self-efficacy mean scores for parents’ education level. The post hoc test revealed that there was a statistically significant difference in self-efficacy mean score between having both parents with higher education and the other groups. Most adolescents reported having support from parents, friends, and teachers. Four-fifths of the adolescents had experienced feeling useful, and just over three quarters had experienced the feeling of mastering things (Table 1).

Based on the examination of self-efficacy across the levels of person factors, social support, and mastery experiences, those who reported good family economy, both parents having higher education, and being male, had statistically significantly higher self-efficacy (person-location estimates of self-efficacy) than their counterparts. No statistically significant differences were observed for the person factor age groups (grade level).

When examining self-efficacy between indicators for social support and mastery experiences, those who reported social support from friends, parents, and teachers, and those who felt useful and felt that they were mastering things, all had significantly higher self-efficacy than those with less social support and fewer mastery experiences.

Table 2 shows the results of the analysis of the association between family economy, parents’ higher education, gender, grade level, mastery experiences, social support, and self-efficacy.

**Table 2:** Association between family economy, parents’ higher education, gender, grade level, social support, mastery experiences and self-efficacy

	**R square**	**Adjusted R square**	**R square change**	**Standardized ** ***β*** ** coefficient**	***p*** **-value**
**Model 1**	0.092	0.091	0.092		
Family economy				-0.213	< 0.001
Parents’ education				0.023	0.042
Gender				-0.196	< 0.001
Grade level				0.026	0.018
**Model 2**	0.144	0.143	0.052		
Family economy				-0.156	< 0.001
Parents’ education				0.014	0.205
Gender				-0.190	< 0.001
Grade level				0.043	< 0.001
*Social support*					
Support from friends				0.111	< 0.001
Parental support				0.135	< 0.001
Support from teachers				0.103	< 0.001
**Model 3**	0.251	0.250	0.107		
Family economy				-0.098	< 0.001
Parents’ education				0.020	0.047
Gender				-0.083	< 0.001
Grade level				0.042	< 0.001
*Social support*					
Support from friends				0.060	< 0.001
Parental support				0.055	< 0.001
Support from teachers				0.023	0.030
*Mastery experiences*					
Felt useful				0.163	< 0.001
Felt that you are mastering things				0.250	< 0.001

This table reports results from sequential multiple regression analysis with self-efficacy as the dependent variable. The independent variables were scored as follows and were entered in the analysis in three sequential steps:

Family economy: 1 = ‘bad’, 2 = ‘nor bad or good’, 3 = ‘bad’

Parents’ education: 1 = no parents with higher education, 2 = one parent with higher education, 3 = both parents with higher education

Gender: 1 = male, 2 = female

Grade level: 1 = 8^th^ grade, 2 = 9^th^ grade, 3 = 10^th^ grade

Social support, friends: 1 = I do not have any nowadays that I can call friends, 2 = I don’t think that, 3 = Yes, I think so, 4 = Yes, definitely

Social support, parents: 1 = not at all true, 2 = not very true, 3 = quite true, 4 = very true

Social support, teachers: 1 = totally disagree, 2 = somewhat disagree, 3 = somewhat agree, 4 =

totally agree

Mastery experiences (‘felt useful’ and ‘felt that you are mastering things’): 1 = never, 2 = seldom, 3 = sometimes, 4 = often, 5 = always

Statistical significance was assumed at *p* < 0.05

After entering the variables: family economy, parents' education, gender, grade, social support, and mastery experiences as independent variables in a sequential multiple linear regression analysis, the variables of the final model (Model 3) explained 25% of the total variance in self-efficacy as the dependent variable (Table 2).

The indicators related to ‘mastery experiences’ explained more of the observed variance in self-efficacy than the other independent variables (change in R square = 10.7% from Model 2 to Model 3; Table 2). The items ‘felt that you are mastering things’ and ‘felt useful’ made the strongest and most significant contributions to the variance in self-efficacy in the final model (β = 0.25, *p* < 0.001 and β = 0.16, *p* < 0.001, respectively), followed by the variables ‘support from friends’ and ‘parental support ‘ (β = 0.06, *p* < 0.001 an β = 0.06, *p* < 0.001, respectively; Table 2). In the final model, bad family economy and being a female also had significant contributions to the variance in self-efficacy (β = -0.10, *p* < 0.001 an β = -0.08, *p* < 0.001, respectively).

## Discussion

This study contributes to the field by examining how mastery experiences and social support are related to self-efficacy. Overall, the main finding is that indicators of mastery experience contribute more to the variance in self-efficacy than other variables. This is in accordance with Bandura’s theory, which holds that personal mastery experiences are especially influential in the development of self-efficacy [10]. Bandura describes these influences as ‘performance accomplishments’.

Few previous studies, if any, have examined factors that strengthen general self-efficacy in adolescents. However, a wide range of previous studies have examined situation-specific self-efficacy, often related to education. For example, results from a study among children aged 8–12 found that mastery experiences did not predict their writing self-efficacy when controlling for the level of writing self-efficacy prior to the intervention [40], which somewhat contradicts our findings. Mastery experiences and positive emotional states have been shown to explain variance in the self-efficacy of pre-school teachers [41], which could be considered more in line with our findings.

In our study, mastery experiences are operationalized by the items ‘felt that you are mastering things’ and ‘felt useful’. As a ‘feeling of mastering things’ has the strongest association with self-efficacy, it is important that adolescents are given tasks that are adapted to their proficiency. Jerusalem and Hessling [14] claim that school is a suitable arena for health promotion and for strengthening adolescents’ self-efficacy. They argue that it is important for students’ academic self-efficacy that task demands are individualised, and that feedback is given on their performance. In addition, a high degree of transparency of teachers’ demands and evaluation criteria is necessary. Teachers need to be clear about their demands, and these demands should be adapted to an individual's skills. This can contribute to adolescents gaining a feeling of mastery. However, adolescents with high self-efficacy may be more motivated to undertake both school-related and practical tasks. Hence, a feeling of mastering things and self-efficacy may be mutually dependent on each other, which is also supported by Bandura [10].

Together with a feeling of mastering things, feeling useful is also positively associated with general self-efficacy in our study. Feeling useful may derive from being a significant person to others, but also from helping others with different tasks. Hence, it may be important for adolescents to contribute to various tasks at home. The aspect of feeling that you are mastering things and feeling useful are potentially highly related. In other words, mastery of an activity may lead to feeling useful, while feeling useful may lead to a sense of mastery. Giving young people both feelings can help them become more robust in facing various challenges they encounter throughout life, since such experiences help to strengthen their self-efficacy.

Our study also shows that adolescents reporting support from friends, parents, and/or teachers had significantly higher mean scores of self-efficacy than those who perceived they had less support. Even though the standardized beta was relatively low, social support also contributed statistically significantly to the variance in self-efficacy. To our knowledge, there are few studies that have explored the association between self-efficacy and social support in adolescents. On one hand, our findings could be deemed as supported by a cross-sectional study among Turkish adolescents that also found an association between peer and family support and academic self-efficacy [42]. However, this study was concerned solely with academic rather than general self-efficacy. Social support was also measured differently than in our study. That said, Schunk and Meece [22] claim that family, school, and peers are social contexts where self-efficacy might be strengthened in adolescence. The present study also shows an association between parental support and self-efficacy, which is supported by Tsang, Hui and Law [43], who highlight the importance of studying the parental role in promoting self-efficacy, as parents play important roles in adolescents’ lives. A review concluded that while 40% of studies on self-efficacy examined teachers, only 2% examined parents [44]. Parents who are available to find tasks and demands that can be adapted to their child, as well as those who give feedback on their performance, can be important for this development. In addition, a good home climate, where parents are sensitive to their children’s needs, is also important.

Jerusalem and Hessling [14] point out that feedback from teachers is important for strengthening adolescent’s self-efficacy. However, in our study, with a lower standardized beta-value than for social support from parents and family and friends, support from teachers can be considered less important for general self-efficacy in adolescents than support from friends and parents.

Our findings indicate that social support is positively associated with self-efficacy. Verbal persuasion, one of the means of developing self-efficacy according to Bandura, might be an indicator for social support. Verbal backup from others – encouraging and believing in the individual could be considered as central aspects of social support. Friends, parents, and/or teachers who support and believe in an adolescent are important in this respect. In addition, friends may also have a positive effect on an individual’s self-efficacy through being social models, which could be linked to what Bandura calls vicarious experiences. To see others who are like yourself, such as friends or fellow students, succeed or master difficult tasks, can have a positive effect on a person’s self-efficacy [22].

Female participants in the present study reported lower self-efficacy than males. These findings are in line with previous cross-sectional studies in China, Germany [45, 46] and Norway [9, 47]. Gender differences in self-efficacy may account for why females experience negative emotions and have lower levels of well-being than males [45]. The gender difference in self-efficacy scores needs to be further explored in future studies, to gain more knowledge on how strategies to promote self-efficacy can be best adapted to different groups of adolescents. Interaction analysis shows that the strength of the association between social support and self-efficacy is not stronger for females compared with males.

Additionally, the current study’s results show a positive association between self-reported family economy and self-efficacy. This positive association may be considered in line with the findings of Mazur et al. [48]. In terms of parental education, however, contradictory results are found. However, Mazur et al. [48] only included mothers’ education as the independent variable in their study. A previous review concluded the need for reduction in socioeconomic inequalities at a societal level to improve mental health in childhood and adolescence [49].

Our final regression model explained 25% of the variance, which means that other factors do also contribute to the variance in self-efficacy. In the light of the theory of Bandura, it is reasonable to assume that factors such as vicarious experience, verbal persuasion, and emotional arousal also may contribute to the variance in self-efficacy, in which should be further studied.

Strengthening self-efficacy in different areas is important in public health settings and is vital to future research. The present findings are also important for guiding health politics. The positive effect of strengthening self-efficacy, presents opportunities for implementing health-promoting interventions; general self-efficacy might be strengthened in any areas where adolescents gain mastery experiences and support. Leisure activities are arenas where adolescents can gain a feeling of mastery of things and being useful. Mastery experiences could also be integrated into school curricula, especially in subjects concerning health issues. However, more studies are needed to explore the effect of general self-efficacy programmes at schools [5]. The present study also indicate that different strategies should be implemented for males and females when promoting self-efficacy, which is in line with a previous study [44].

### Strengths and limitations

The main strength of this study is a large sample size and a high response rate. This study also extends the perspectives of many previous studies, by not looking at self-efficacy solely as an independent variable or a moderator. However, the analyses are based on self-reported data; hence, there may be a risk of response bias (e.g. social desirability). Nevertheless, considering the large sample size of this study and the fact that the questionnaire was completed anonymously, potential random errors are minimised. The data was collected during the second year of the Covid-19 pandemic, so there is a possibility that this might have affected the answers.

Data used in this study is cross-sectional, meaning that no causal conclusions can be drawn. Hence, that there is a possibility that adolescents with stronger self-efficacy experience mastery and the feeling of being useful more easily – and experience greater support from friends, family, and teachers. However, Bandura’s theory on self-efficacy supports our analyses, and our suggested main direction on the relationship between mastering things, feeling useful, and self-efficacy.

There is also a possibility that the questions included in this study are perceived differently by different adolescents. At the same time, feelings of mastery or being useful are to a great degree subjective. It is possible that two adolescents objectively may share the same experience and perform the same task, but that their experience differs in terms of mastery. Therefore, the individual perspective and individual adaption are of high importance, for example when developing self-efficacy interventions.

Bandura [10] describes four factors related to the development of self-efficacy. In this study, we have not studied Bandura’s theory in greater depth, as we only have included those variables available in the Municipal Youth Surveys that are related to mastery experiences and social support. Hence, the extent to which the variables we have included can be related to the four factors described by Bandura is open to discussion. Future studies should focus on other relevant theoretical aspects of self-efficacy, such as the impact of the mastery experiences of friends and schoolmates.

## Conclusion

Mastery experiences, such as the feeling of mastering things and being useful, are found in this study to be associated with self-efficacy in adolescents. The same is true of social support. Consequently, it can be considered as important that significant adults help prepare adolescents for mastery experiences, whether at school, at home, or in leisure activities. Mastery experiences and social support are important factors for strengthening self-efficacy, which may in turn have a positive impact on the mental health of adolescents. There is a presumably concern about mental health among adolescents. In order to address this challenge, policymakers should take a more health-promoting perspective, emphasizing factors that strengthen adolescents' mental health and increasing their knowledge about what potentially strengthens self-efficacy. Future studies should further explore how mastery experiences, social support, and self-efficacy can be implemented in health-promoting strategies for adolescents both at school, at home, and in their leisure time. In addition, more knowledge is needed on the impact of the other components of Bandura’s theory to strengthening self-efficacy in adolescents.

## Data Availability

The datasets generated and analysed during the current study are not publicly available due to that the data and materials from the Ungdata Surveys are protected and stored in a national database administered by NOVA. The data are available for research purposes upon application from the ungdata@oslomet.no, and is not available from the corresponding author. Further information about the study and the questionnaires can be found on the web page (in Norwegian or English) (http://ungdata.no/ or https://www.ungdata.no/english/).
